# Bridging Dynamical Systems and Optimal Trajectory Approaches to Speech Motor Control With Dynamic Movement Primitives

**DOI:** 10.3389/fpsyg.2019.02251

**Published:** 2019-10-14

**Authors:** Benjamin Parrell, Adam C. Lammert

**Affiliations:** ^1^Department of Communication Sciences & Disorders, University of Wisconsin-Madison, Madison, WI, United States; ^2^Department of Biomedical Engineering, Worcester Polytechnic Institute, Worcester, MA, United States; ^3^Bioengineering Systems & Technologies, MIT Lincoln Laboratory, Lexington, MA, United States

**Keywords:** speech motor control, computational models, dynamical systems, optimal control, task dynamics, dynamic movement primitives

## Abstract

Current models of speech motor control rely on either trajectory-based control (DIVA, GEPPETO, ACT) or a dynamical systems approach based on feedback control (Task Dynamics, FACTS). While both approaches have provided insights into the speech motor system, it is difficult to connect these findings across models given the distinct theoretical and computational bases of the two approaches. We propose a new extension of the most widely used dynamical systems approach, Task Dynamics, that incorporates many of the strengths of trajectory-based approaches, providing a way to bridge the theoretical divide between what have been two separate approaches to understanding speech motor control. The Task Dynamics (TD) model posits that speech gestures are governed by point attractor dynamics consistent with a critically damped harmonic oscillator. Kinematic trajectories associated with such gestures should therefore be consistent with a second-order dynamical system, possibly modified by blending with temporally overlapping gestures or altering oscillator parameters. This account of observed kinematics is powerful and theoretically appealing, but may be insufficient to account for deviations from predicted kinematics—i.e., changes produced in response to some external perturbations to the jaw, changes in control during acquisition and development, or effects of word/syllable frequency. Optimization, such as would be needed to minimize articulatory effort, is also incompatible with the current TD model, though the idea that the speech production systems economizes effort has a long history and, importantly, also plays a critical role in current theories of domain-general human motor control. To address these issues, we use Dynamic Movement Primitives (DMPs) to expand a dynamical systems framework for speech motor control to allow modification of kinematic trajectories by incorporating a simple, learnable forcing term into existing point attractor dynamics. We show that integration of DMPs with task-based point-attractor dynamics enhances the potential explanatory power of TD in a number of critical ways, including the ability to account for external forces in planning and optimizing both kinematic and dynamic movement costs. At the same time, this approach preserves the successes of Task Dynamics in handling multi-gesture planning and coordination.

## Introduction

The speech motor system comprises many individual subsystems (respiratory, phonatory, articulatory), a larger number of individual articulators (upper lip, lower lip, jaw, tongue tip, tongue body, etc.), and an even larger number of muscles. The highly redundant structure of this system ensures that there are often many (perhaps infinite) ways for the system to move between two given configurations (Bernstein, 1967). How are speakers able to select from among the multitude of possible movement patterns, to arrive at those representing the highly accurate and precise movements that typify healthy, mature speech? Attempts to explain the speech control systems that produce such complex behavior have fallen into two opposing approaches: (1) dynamical systems theory, which conceptualizes movement patterns as emergent properties of synergistic groups or systems of speech articulators whose evolution is determined by the state of the system and current production goals, and (2) trajectory-based approaches, which solve the highly redundant control problem by pre-specifying a particular desired trajectory. A subset of this latter approach which will be particularly relevant to the current proposal are optimality-based approaches, which attempt to find a desired trajectory that minimizes some cost function (either kinematic properties of the movement, such as jerk, or dynamic properties, such as total force). While both dynamical systems and optimal control approaches have had success in replicating certain aspects of human speech behavior, they have arrived at essentially distinct understandings of the nature of speech motor control.

The dynamical systems approach suggests that control of the complex motor system can be considerably simplified by understanding the motor system as a self-organizing system of functional units of articulators, each of which corresponds to a particular behavioral task. The behavior of the component articulators, while governed by the higher-order functional unit, need not be explicitly or directly specified. These functional units thus serve to constrain the motor system in such a way that its evolution serves to perform the particular task specified by the functional unit, without the need to centrally control the activity of each degree of freedom in the system. Practically, these functional units are hypothesized to be autonomous dynamical systems whose evolution depends on the system's current and goal states. The particular parameters of each dynamical system (e.g., the goal, stiffness, damping, etc.) govern the evolution of the system from its current state toward a goal state, and the evolution of this dynamical system generates the motor activity in the lower-level subsystems needed to perform the task. Importantly, there is no specific plan or desired kinematic trajectory in this approach. Instead, the kinematic behavior of the system emerges from the dynamical regime governing the functional unit (which could alternatively be called the controller).

The most prevalent dynamical systems model of speech production is the Task Dynamic Model^1^ (Saltzman, 1986; Saltzman and Munhall, 1989). In this theory, speech tasks are modeled as a second-order, damped mass-spring systems. The evolution of such a system is given by Equation (1) (discussed in more detail in the Task Dynamics section).

(1) $$\begin{array}{l} \ddot{z}=M^{-1}\left(-B\dot{z}-K\left(z-g\right)\right) \end{array}$$

Where $\ddot{z}$ is the system acceleration of the system state, *z* is the current position, $\dot{z}$ is the current velocity, *g* is the target spatial position or goal, and *M, B*, and *K*, respectively, the mass, damping, and stiffness coefficients, which are assumed to reflect critical damping. Such systems have two desirable characteristics for a motor controller. First, they exhibit equifinality, such that the system will come to rest at its target position regardless of the initial state of the system. This also assures that the system will reach its resting position regardless of any perturbations that may occur during the movement without the need for any re-planning or change in control. Second, such systems are time-invariant, in that the evolution of the system is a function governed by its current state and dynamical parameters (spatial target, mass, stiffness, damping) rather than being explicitly a function of time. This is a particularly important consideration for speech, where the duration of individual movements is affected by a wide range of parameters, including speech rate, stress, and prosodic structure.

Dynamical approaches to movement control receive some support from research on neurobiological control systems. For example, the VITE model (Bullock and Grossberg, 1988) presents a relatively simple neural network model that is able to generate appropriate kinematic behavior in directed reaching movements. The model consists of three distinct but interacting neural populations encoding (1) the present position of the system, (2) the desired target position, and (3) the difference between the target and present positions. The relational structure between these populations is such that the behavior of the controlled systems is consistent with second-order dynamics. This suggests a plausible neural implementation of the more abstract dynamical systems in Task Dynamics (Lammert et al., 2018). Additionally, recent studies have identified dynamical patterns in the neural activity that drives motor behavior (Churchland et al., 2012; Shenoy et al., 2013). Using intracortical recordings in non-human primates, these studies have shown that oscillatory motor behavior, such as walking, is reflected at a neural level by co-occurring oscillatory dynamics at the population level in the activity of motor cortical neurons. Importantly, cortical activity during goal-directed reaching, a non-oscillatory behavior, also exhibits patterns of neural activity consistent with a truncated limit-cycle oscillator. These results have recently been extended to human speech, where similar dynamical patterns have been demonstrated in the population-level activity of primary motor cortex neurons during production of monosyllabic words (Stavisky et al., 2018). Together, these results suggest that a controller based on dynamical equations may be an appropriate model of the neural implementation of motor control.

The principal drawback of the Task Dynamics implementation of dynamical systems control is that the dynamics driving the evolution of the functional task units are limited in flexibility. The system is only able to generate oscillatory dynamics (with various degrees of damping), such that the system will evolve in a deterministic way from any given initial state toward the goal state. Though these movements can, in principle, be modified in a potentially profound way by changing the damping, stiffness and inertial coefficients, such changes would only globally affect each gesture within the system.

Extensions of Task Dynamics have attempted to address this limitation for specific cases. Prosodic gestures have been proposed that allow for temporally-specific changes in the rate and/or extent of movements (Byrd et al., 2000; Byrd and Saltzman, 2003; Saltzman et al., 2008), though these prosodic gestures act concurrently on all active gestures, rather than specifically on individual gestures. Also, multiple gestures produced with varying degrees of temporal overlap have been shown to result in movements that are truncated and forced to reverse direction prematurely, which can account for reduction phenomena, such as undershoot, flapping, and spirantization (Browman and Goldstein, 1990, 1992; Edwards et al., 1991; Beckman and Edwards, 1992; Beckman et al., 1992; Parrell, 2011; Parrell and Narayanan, 2018).

Despite these important modeling advances, the Task Dynamics implementation of dynamical systems control is still unable to produce local changes in the rate of change or reversals of direction arbitrarily, or for any single activated tract-variable (TV) gesture. While such behavior may not be critical for some aspects of speech (see the large literature on modeling speech using second order dynamics), some speech behaviors do require more complex control. For example, when speakers are exposed to a velocity-dependent force field on the jaw, they initially produce jaw trajectories that deviate, or curve away, from the relatively straight trajectories observed under unperturbed conditions (Tremblay et al., 2003, 2008; Tremblay and Ostry, 2006; Lametti et al., 2012). However, after a period of exposure, jaw trajectories return to their baseline curvature. When the force field is subsequently removed, jaw trajectories are curved in the opposite direction as under initial exposure. These results suggest that the speech motor control system can learn to account for the dynamics of the force field to generate motor commands that maintain a straight trajectory. Moreover, some have argued that speech motor control may rely on explicit trajectory representations rather than discrete attractors (Guenther, 2016) or that the speech motor system seeks to balance effort and intelligibility (Lindblom, 1990; Perrier et al., 2005; Patri et al., 2015). These types of behavior cannot be generated in Task Dynamics or any control system whose dynamics are dependent only on the system state.

In order to account for behaviors exhibited by speakers in the jaw perturbation paradigm discussed above, the controller must be sensitive to other types of information beyond the instantaneous system state. One solution to this problem is found in theories that rely on optimization to generate motor output. Such schemes, known as optimal controllers, seek to generate a movement that minimizes some cost function. Typically, this involves the generation of a pre-planned motor trajectory, such that the cost of the full movement can be calculated and minimized prior to movement onset [though see optimal feedback control, e.g., Todorov and Jordan (2002), for a variation of optimal control without pre-planned trajectories].

Optimal control has a long history in modeling discrete reaching tasks (Nelson, 1983; Flash and Hogan, 1985; Uno et al., 1989; Hoff and Arbib, 1993; Harris and Wolpert, 1998) as well as in speech (Perrier et al., 2005; Patri et al., 2015). While these models share the general concept of optimizing movements to minimize some cost, the nature of the cost function has been a matter of debate. It is often claimed that the central nervous systems minimizes the total muscle activation of a movement (Harris and Wolpert, 1998; Todorov and Jordan, 2002; Todorov, 2004; Perrier et al., 2005; Patri et al., 2015), either to minimize the amount of energy expended during a movement or to minimize error. Error is minimized along with total muscle activation because noise in the motor system is signal dependent, such that the variance of force scales proportionally with the square of the force (O'Sullivan et al., 2009; Diedrichsen et al., 2010). Other proposals suggest that the kinematic characteristics of movement determine the cost function. Cost functions have been suggested to minimize jerk, which is the third derivative of position (Flash and Hogan, 1985; Hoff and Arbib, 1993), torque change (Uno et al., 1989), or path curvature (Kistemaker et al., 2010, 2014). Regardless of their specific implementation, such proposals are able to account for external as well as internal dynamics in control, and are able to produce changes in behavior in response to force field perturbations (Izawa et al., 2008).

In speech, optimal control has been implemented in the GEPPETO (Perrier et al., 2005) model and its Bayesian reformulation (Patri et al., 2015). It is also, implicitly, incorporated into DIVA (Guenther, 2016). DIVA differs from many optimal control approaches in that it attempts to optimize planned motor trajectories with respect to a given reference (sensory) trajectory. Optimization serves the purpose of accurately following the reference trajectory, rather than minimizing some criterion intrinsic to the planned trajectory itself, such as effort. This is accomplished by summing, over time, corrective motor commands issued by the auditory and somatosensory feedback controllers, which can be seen as a type of iterative optimization.

Most optimal control models, including those of speech, rely on the generation of movement trajectories. This is partly because identifying specific, optimal trajectories is more computationally tractable when compared to identifying more general optimal control policies (Schaal et al., 2007). Trajectories (or, more precisely, time-varying targets) have also been suggested to be necessary for speech (Guenther, 2016). Trajectory-based control can also substantially simplify the degrees-of-freedom problem if trajectories are planned in mobility space^2^ (as occurs in DIVA and GEPPETO), since each degree of freedom is explicitly accounted for. However, trajectories lack flexibility, and may require frequent replanning/reoptimization in the face of changing environments or task demands. Trajectory-based control is also inherently time-indexed, in that trajectories are defined as a function of time. Such time-indexing has strict consequences for the validity of trajectory-tracking control policies in changing environments, and may also be difficult to reconcile with the temporally malleable speech production system (e.g., movement durations are affected by speech rate, stress, prosodic boundaries, etc.). Moreover, trajectory-based optimal controllers make inaccurate predictions about the types of variability observed in human kinematics (Todorov and Jordan, 2002). And, perhaps most importantly, there is growing evidence that human movement does not rely on fully pre-planned trajectories, at least for limb control (Sergio and Scott, 1998; Desmurget and Grafton, 2000; Nashed et al., 2012).

Thus, the field of speech motor control is left with a situation where neither the dynamical systems nor optimal control approaches provide fully satisfactory accounts of human motor behavior. An ideal control system would provide the flexibility, temporal flexibility, and robustness of the dynamical systems approach with the ability to account for the behavioral evidence that humans do produce motor behavior in accordance with particular dynamic and/or kinematic constraints.

A few approaches in human motor control and robotics have sought to bridge this divide. These include Optimal Feedback Control (Todorov and Jordan, 2002; Todorov, 2004), Dynamic Movement Primitives (Schaal et al., 2007; Ijspeert et al., 2013), and Embodied Task Dynamics (Simko and Cummins, 2010a,b, 2011). Optimal Feedback Control (OFC) replaces trajectory-based optimization with an optimal feedback control law. While this solves many of the issues with traditional optimal control, the derivation and calculation of this optimal feedback control law is difficult, especially for non-linear systems like speech. The approach based on Dynamic Movement Primitives (DMPs) incorporates an additional *forcing function* into a second order dynamical control system that can be tuned to alter the trajectory produced by the dynamical control system. This approach is substantially easier to compute and, perhaps more importantly, retains the many benefits provided by existing dynamical control schemes. Embodied Task Dynamics is an extension of Task Dynamics that incorporates the physical masses of the speech articulators into the equations of control. This allows for the quantification of effort (sum of forces), which is then used in a cost function along with constraints on movement duration and speech intelligibility.

The current paper presents a step toward bridging the substantial theoretical gap that separates dynamical systems and optimal or trajectory-based approaches to speech motor control. We accomplish this by leveraging the tools of Dynamic Movements Primitives (Ijspeert et al., 2013) to incorporate optimization into the most well-developed dynamical-systems framework of speech motor control, Task Dynamics. In the sections below, we lay out the basics of dynamical control in Task Dynamics, DMPs, and the coordination of DMPs with second-order dynamical systems. We then demonstrate the utility of this combined model by showing how this approach can be used to generate corrections for dynamic jaw perturbations that are consistent with experimentally measured human behavior. Lastly, we show how the mechanisms developed to incorporate DMPs into second-order dynamical systems can also be used as a system of intergestural coordination (Nam and Saltzman, 2003; Saltzman et al., 2008; Goldstein et al., 2009) as well as movement initiation (Tilsen, 2013).

## Task Dynamics Model

Articulatory Phonology (AP) posits that constriction actions (i.e., gestures) of the vocal tract represent both the primitive units of spoken language and the controlled tasks that characterize speech motor control (Browman and Goldstein, 1992). The Task Dynamics (TD) model asserts that the controlled evolution in time of these constriction actions is governed by second-order equations of motion, consistent with a critically damped harmonic oscillator.

Speech gestures and their associated dynamics take place in a space described by a vector of N *tract variables*, *z*, where *z* = [*z*_1_, *z*_2_, …, *z*_*N*_], that correspond to the degree and location of vocal tract constrictions. Each specific gesture, *k*, is associated with its own pair of constriction degree and location tract-variables and its own set of mobility variables. Additionally, each gesture is associated with a corresponding set of tract-variable dynamic parameters (spatial target, mass, damping, and stiffness, all time-invariant) and articulator weights. Articulator weights are described below in conjunction with Equation 6. Gestures themselves are governed by equations of motion consistent with a damped harmonic oscillator, as described by Saltzman and Kelso (1987) and Saltzman and Munhall (1989):

(2) $$\begin{array}{l} M\ddot{z}=-B\dot{z}-K\Delta z \end{array}$$

where Δ*z* = (*z* − *g*), and *g* is a vector containing the time-varying set of parameters representing the current set of tract-variable spatial motor goals—i.e., the target positions to which the tract variables are compelled to move and upon which they will tend to converge. *M*, *B*, *K* are diagonal matrices containing the mass, damping, and stiffness coefficients, respectively. All tract variable parameters, *M*, *B*, *K*, and *g*, change over time as functions of the currently active set of gestures. As noted above, the stiffness, damping and inertial gestural parameters can have a profound influence on the gesture-related movement trajectories. These parameters, from a broader perspective, may therefore be considered part of the motor goals of the system, e.g., stiffness parameters are lower for vowels than consonants to capture the fact that vowel gestures are typically slower than consonant gestures.

The TD model also defines the relationships between the tract variables and relatively lower-level mobility variables, ϕ. Tract variables describe the state of the vocal tract with respect to speech gestures. However, the vocal tract, like many motor systems, is typically considered to have a hierarchical structure, where motor goals are defined in a high-level *task space*, and motor commands are issued in a low-level *mobility space*. For example, in a speech context, mobility space variables might be expressed in terms of the positions of the speech articulators (e.g., upper lip, lower lip, tongue tip, etc., called the *model articulators* in TD), or even in terms of muscle activations^3^. The relevant kinematic equations that define the relationships between the task and mobility spaces are expressed as follows:

(3) $$\begin{array}{l} z=h\left(\phi \right), \end{array}$$

(4) $$\begin{array}{l} \dot{z}=J\left(\phi \right)\overset{∙}{\phi }, \end{array}$$

(5) $$\begin{array}{l} \ddot{z}=\left(\phi \right)\ddot{\phi }+\overset{∙}{J}\left(\phi ,\overset{∙}{\phi }\right)\overset{∙}{\phi } \end{array}$$

where *h* represents the direct kinematic mapping between task and mobility spaces, and *J* is the Jacobian matrix of first-order partial derivatives of *z* with respect to ϕ.

Using these kinematic relationships, one can express accelerations of the controlled, mobility space variables with respect to the task-space error:

(6) $$\begin{array}{l} \ddot{\phi }=J^{*}\left(M^{-1}\left[-BJ\overset{∙}{\phi }-K\Delta z\right]\right)-J^{*}\overset{∙}{J}\overset{∙}{\phi }, \end{array}$$

where J^*^ = *W*^−1^*J*^*T*^(*JW*^−1^*J*^*T*^)^−1^ is the pseudo-inverse of the Jacobian, weighted by a matrix *W*. The equation of motion, in Equation (6), for mobility space variables represents the full expression of the dynamical control law that characterizes TD, with integrated inverse kinematics, specifying how task-space error is equated to a preferred change in mobility space. It is worth noting that the weighted Jacobian pseudo-inverse provides a minimum norm solution that can be considered optimal in the sense that it minimizes the weighted sum of squared mobility-space accelerations selected for the solution. As evidenced by this fact, it is possible to incorporate some aspects of preferred optimality directly into a dynamical systems control algorithm.

In Task Dynamics, the activation of a gesture is determined by its associated planning oscillator, a second order dynamical system with non-linear damping. The activation of a gesture is determined by the phase of this planning oscillator. Essentially, the phase of the oscillator determines the value of the “go” signal (*G*), which allows motion associated with a gesture to proceed. Early versions of Task Dynamics used a step function to define this relationship—e.g., *G* = 1 while the planning oscillator phase is between 0 and 270°). More recent versions have used a cosine-ramped activation function, which results in more realistic kinematics (Byrd and Saltzman, 1998).

Note that accelerations are potentially experienced by all mobility variables, even those that are not engaged by currently-active gestures, due to the inclusion of a neutral attractor. The neutral attractor amounts to a mobility-space target position that drives mobility variables in the absence of driving influences from currently-active gestures.

**Figure 1:**
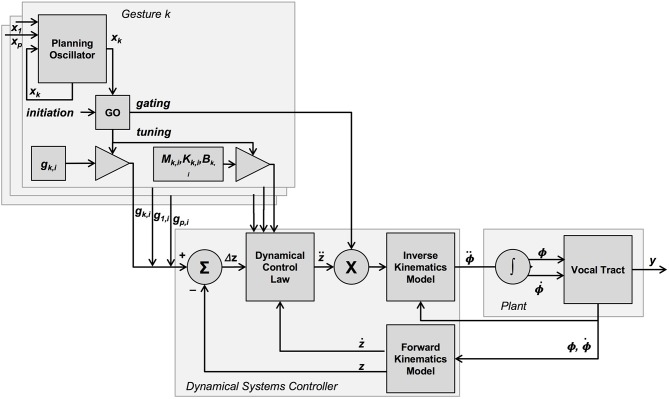
Graphical overview of the TD model, as presented in Saltzman and Munhall (1989) and Byrd and Saltzman (1998). The dynamical systems controller takes, as input, gesture-specific sets of parameter values (task-space target, as well as inertial, damping and stiffness parameters) for all active gestures, and determines via forward dynamics (dynamical control law) a corresponding set of accelerations for these active tract-variables. This set of active tract-variable accelerations is then gated into a corresponding set of mobility-variable accelerations via inverse kinematics for those mobility variables associated with the active tract-variables. All gesture-specific parameters are gated by the “go” signal, which is a function of a gesture initiation signal as well as the value of a gesture-specific planning oscillator. This oscillator is potentially coupled to the planning oscillators of other gestures.

The “go” signal itself is incorporated into TD in the form of a gating matrix, included as part of the inverse kinematics model (Saltzman and Munhall, 1989)^4^, as well as a gesture-specific parameter tuning function (spatial target *g* as well as damping and spring coefficients—all mass coefficients have been set to 1 for simplicity) for the dynamical control law (see Figure 1). Note that the role of the “go” signal used in gesture tuning is similar to and consistent with other models of directed action—e.g., Bullock and Grossberg (1988).

## Dynamic Movement Primitives

The general idea of Dynamic Movement Primitives (DMPs) is to augment a dynamical systems model, like that found in Equation (2), with a flexible forcing function input, *f*. The addition of a forcing function allows the present model to overcome certain inflexibilities inherent in the original TD model. Given a speech gesture—conceptualized in AP and TD as comprising a set of a constriction target and inertial, damping and stiffness parameters—and a set of initial conditions, the unforced patterns of movement in TD are entirely determined by Equation (2). Without some method of otherwise influencing the dynamics, a speech gesture under the same initial conditions will follow the same pattern of movement during each instance of that gesture. Conversely, if the system is subjected to some external perturbation, the changes in movement associated with that perturbation will persist indefinitely. The addition of the forcing term allows for flexible modification of the trajectories of the tract variables as they move toward the spatial motor goal, all while preserving the dynamical form of the TD model. A forcing term of this type, and for this purpose, has been suggested and developed by Ijspeert et al. (Ijspeert et al., 2002, 2013; Hoffmann et al., 2009).

We refer to the dynamic control law augmented with a flexible forcing function input as the *control system*. In order for this forcing function to flexibly alter the evolution of the dynamical system, it must be time-variant. However, if the forcing function is explicitly a function of time—i.e., *f(t)*—such a formulation would remove one of the key benefits of dynamical systems control, which is that they are time invariant. To avoid this, we replace any explicit time dependency with a dependency on a separate dynamical system, the *planning system*, *f*(*x*). In the sections that follow, we first describe the nature of the control system and forcing function, then discuss details of the planning system^5^.

### Control System

In the control system, the dynamical systems model in Equation (2) is augmented with a forcing function input, *f*, as follows:

(7) $$\begin{array}{l} M\ddot{z}=-B\dot{z}-K\Delta z+f \end{array}$$

The forcing term is a vector of forces acting on the vocal tract dynamics, where each element is also associated with a specific tract variable and specific gesture over that tract variable.

For a specific gesture *k*, the forcing term *f*_*k*_, an input to the control system, is a function of the planning system state, *x*, with the following form:

(8) $$\begin{array}{l} f_{k}\left(x_{k}\right)=\;\frac{\sum_{j=1}^{n}{\text{Ψ}}_{j}\left(x\right)w_{j}}{\sum_{j=1}^{n}{\text{Ψ}}_{j}\left(x\right)}\left(\frac{2\pi -x\text{mod}2\pi }{2\pi }\right)\left(g_{k}-z_{0}\right) \end{array}$$

where *z*_0_ is the initial state of the tract variable associated with the gesture. Thus, the forcing term is essentially a linear combination of *n* fixed kernel functions Ψ_*j*_, each of which are a function of the planning system state and scaled according to kernel-specific weights *w*_*j*_. Because the planning system will be defined to converge to 2π, scaling this weighting by (2π − *x*mod2π)/2π ensures that the overall forcing function will tend toward zero as the planning system converges. This, in turn, ensures that the control system will converge to zero, eventually, as the dynamics revert to that of a damped spring-mass system. The purpose of scaling by *g*_*k*_ − *z*_0_ is to ensure certain advantageous invariance properties when scaling movements, as outlined by Ijspeert et al. (2013). We will not treat these invariance properties in depth in the current discussion.

The kernel functions have an exponential form:

(9) $$\begin{array}{l} {\text{Ψ}}_{j}\left(x\right)=\text{exp}\left(-\frac{1}{2\sigma_{j}^{2}}{\left(x-c_{j}\right)}^{2}\right) \end{array}$$

giving them a Gaussian shape, with a specific kernel center *c*_*j*_ that situates the kernel center relative to some planning system state, and also defined by a kernel width parameter σ_*j*_. As the planning system state evolves, kernel functions that are centered on specific state values will become more highly weighted, to the point where their centers align exactly with the planning system state, and subsequently become less weighted as the planning system evolves beyond that point. As pointed out by Ijspeert et al. (2013), this has similarities with vector-coding models of neural activation.

**Figure 2:**
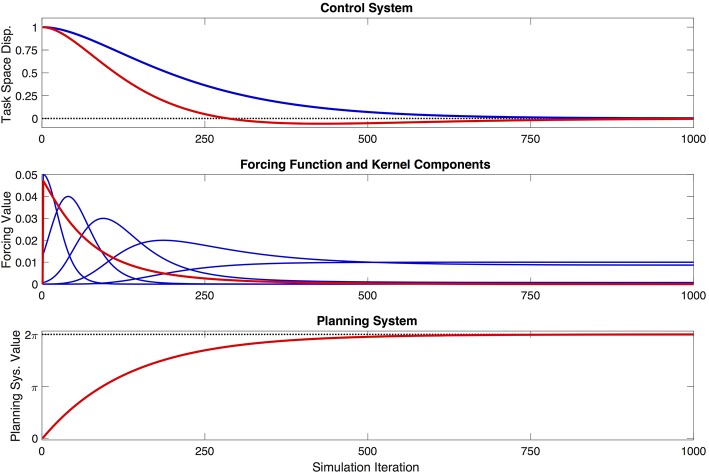
Example behavior of the proposed model, showing the model's evolution over the course of one simulation. The one-dimensional control system is initialized with a displacement of 1 task-space unit, and aims to reach a target displacement of 0. The top figure shows the movement of the control system (red) in task space, the shape of which is being partially determined by a forcing function, and is therefore modified relative to a generic, unforced dynamical system (blue) with identical parameters. The middle figure shows the shape of the forcing function (red), and the five weighted kernel functions (blue) that contribute to that shape. Note that the kernels appear asymmetrical in the time domain—i.e., simulation iterations—because they are defined as symmetrical in the planning variable, *x*. The bottom figure shows the evolution of the planning system (red) as it completes one cycle, and converges to a value of 2π.

Several aspects of the model related to the kernel functions are worth noting. First, the kernels as implemented are defined as symmetrical in the planning system domain, *x*, which means that they are not necessarily symmetrical in the time domain. This can be clearly seen in Figure 2. Second, the degree of flexibility afforded to the control system via the kernels—insofar as they are used to compose the forcing function that directly influences the control system—will depend on the number of kernels used, their spacing in *x*, and the width parameter σ associated with each kernel. In broad terms, more flexibility will be associated with more, narrower kernels that are more closely spaced. Increased flexibility comes, however, at the expense of parsimony of the model. The tradeoff between flexibility and parsimony is an interesting one, the solution to which will certainly be application-specific, and could even be determined as part of an optimization process. For present purpose, it is assumed that the number, spacing, and width of the kernels is fixed. Following previous presentations of DMPs (Schaal et al., 2007; Ijspeert et al., 2013), we leave the question of the optimal kernel parameterization open for future work.

### Planning System

To coordinate the activation of kernel functions in conjunction with a specific gesture, it is helpful to define a *planning system* for that gesture. Importantly, the use of a planning system also allows the control system to be abstracted away from linear time dependency. The planning system comprises a first-order dynamical system of the following form:

(10) $$\begin{array}{l} m_{i}{\dot{x}}_{k}=\alpha_{x}\left(2\pi -x_{k}\text{mod}2\pi \right). \end{array}$$

The state of this system is *x*_*k*_, the constant α_*x*_ determines the rate of convergence, and *m*_*i*_ is a tract variable-specific inertial parameter, a component of *M* from above. For present purposes, it is assumed that this system is initiated, at the beginning of a discrete gesture, with a value of 0. The dynamics of the planning system will cause it to subsequently converge to the next multiple of 2π, completing one full cycle^6^.

**Figure 3:**
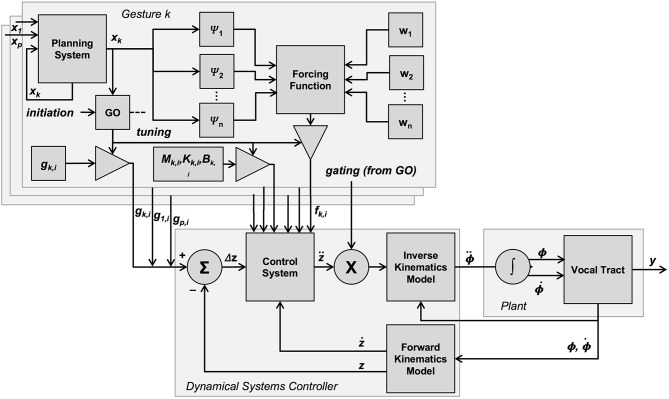
Graphical overview of the present model. In this model, many properties of the TD model are preserved, with the main difference being the addition of mechanisms surrounding the generation of the forcing function. The forcing function influences the control system. It is generated on the basis of several kernels, which are a function of the planning system state, as well as weighting values assigned to each kernel. Like the targets, the forcing function associated with each gesture is gated by the “go” signal, and the value of a gesture-specific planning system, which is potentially coupled to the planning system of other gestures.

The planning system serves two purposes. First, the evolution of the system's state also serves as the basis for activating the primitive kernels at the appropriate time during that gesture. Second, the planning system can also be used to define the “go” signal, which allows motion associated with a gesture to proceed. For present purposes, we define the “go” signal as a rectangular step function of the planning system state:

(11) $$\begin{array}{l} G_{k}=\;\left\{ \begin{array}{cc} 1\cdot I, & if\;0+\varepsilon <\;x_{i}\text{mod}2\pi <2\pi -\varepsilon \\ 0, & otherwise \end{array}\right. \end{array}$$

where ε ≈ 0 and *k* is the gesture. As shown in Figure 3, the “go” signal gates the inclusion of a gesture-specific target *g* into the vector of currently-active targets, similar to its function in the original TD model (see Figure 1), as well as the inclusion of other gestural parameters into the dynamical control law. In the present model, the “go” signal also gates the contribution of the forcing function *f* to the control system.

The “go” signal *G* is also modulated by an initiation signal, which is the results of a higher-level process monitoring an initial planning phase, during which the several (perhaps coupled) planning systems associated with an utterance are allowed to oscillate and converge to a stable temporal coordination pattern (see below for an extended example). Before convergence, the value of *I* is set to 0 and, after convergence, the value of becomes 1, and remains at that value until the entire utterance is complete. This change in value has the effect of allowing the movement associated with some gestures to commence, in accordance with the coordination pattern converged upon during the planning period. A similar initiation signal must be present in the planning oscillator formulation of Task Dynamics to drive the switch from planning to action.

Note that the function defined in Equation (11), above, might be most appropriately cast as another kernel function, which would be consistent with the use of kernel functions in the present framework, and which would allow for continuous rise and fall times, consistent with the gestural activations presented in the TD framework (Byrd and Saltzman, 1998; Saltzman, 1999).

## Kernel Weight Estimation and Movement Optimization

Having established the general form of the forcing function and planning system, we move to a discussion of how the weights of the kernels in forcing function can be assigned. Importantly, this is where optimization is incorporated into the model. While kernel weights could, in theory, be assigned to achieve any goal, in practice we show how the weights can be assigned to minimize some movement cost, following optimal control approaches. We take an agnostic stance over what aspect of movement may be optimized: there is evidence that both kinematic (Flash and Hogan, 1985; Uno et al., 1989; Hoff and Arbib, 1993; Kistemaker et al., 2010, 2014; Mistry et al., 2013) and dynamic properties (Todorov and Jordan, 2002; Todorov, 2004; Izawa et al., 2008; Diedrichsen et al., 2010) of movement may serve this function. In the following sections, we first show how DMPs may be used to minimize a kinematic constraint (trajectory tracking or straightness) as well as a dynamic constraint (effort minimization). We then show how both approaches are able to replicate the behavior of human speakers exposed to velocity-dependent force fields applied to the jaw during speech.

### Trajectory Tracking Optimization

One approach to assigning the kernel weights is to do so such that some reference trajectory is accurately reproduced. If a specific trajectory shape is desirable, e.g., a straight line (Kistemaker et al., 2010), it is possible to compute a set of weights that will approximate that shape to the extent possible given the number and spacing of kernel functions available. Computing the weights requires an inversion of the control system dynamics, with environmental effects taken into account, in order to find the forcing function, which is what must be approximated by the weighted kernel functions.

With a detailed internal model of the dynamics of both the body and the environment, an estimate of the forcing function can be estimated. This can begin with Equation (7), accounting for the DMP-related forcing term (*f*_*s*_), as well as any additional forces (*f*_*p*_), due to environmental influences (e.g., perturbations). If one has a reference trajectory measured as a function of time, *z*_*ref*_(*t*), the dynamics can be directly inverted, leading to the estimate:

(12) $$\begin{array}{lll} f_{s}\left(t\right) & = & (M{\ddot{z}}_{ref}\left(t\right)-K\left(z_{ref}\left(t\right)-g\right)+B{\dot{z}}_{ref}\left(t\right)\\ \; & \; & +f_{p}\left(t\right))/\left(g-z_{ref}\left(0\right)\right) \end{array}$$

This estimate of the forcing function can be used to form an estimate of the kernel weights. Because the kernels are a function of the planning system (*x*) and not time (*t*), this first requires that the planning system be integrated, providing an estimate of the planning system as a function of time, *x*(*t*). Finally, linear regression can be used to solve for the weights, given the known shape of the kernel functions, using these time functions. This general procedure was outlined by Hoffmann et al. (2009).

### Minimum Effort Optimization

Many possible approaches exist to optimizing a function based on the system output. One approach is to optimize the accumulated effort associated with a movement by minimizing it. Minimum-effort optimization criteria have a long history in models of motor control (Nelson, 1983; Todorov and Jordan, 2002; Todorov, 2004; Perrier et al., 2005; Patri et al., 2015), and minimal-effort criteria have been suggested to play an important role in speech production (Lindblom, 1990). DMPs afford the necessary flexibility to optimize dynamical systems control in this way. We provide an example of an iterative approach to effort minimization, using a simple method of updating the kernel weights, over many instances of a movement, based on an effort calculation. While more complicated optimization algorithms could be used, this straightforward iterative approach is used here as a proof of concept.

Admitting that, due to stochastic factors, such as those associated with neural activity, no two repetitions of any action will be precisely the same, a small extension of Equation (8) can be made, as follows:

(13) $$f_{k}\left(x_{k}\right)=\;\frac{\sum_{j=1}^{n}\Psi_{j}\left(x\right)[w_{j}+\varepsilon N(\mu ,\sigma^{2})]}{\sum_{j=1}^{n}\Psi_{j}\left(x\right)}\left(\frac{2\pi -x}{2\pi }\right)(g_{k}-z_{0}),$$

for some small value of ε, and where $N\left(\mu ,\sigma^{2}\right)$ is the normal distribution with mean μ and variance σ^2^. Deviations in the controlled forces implied by this change will likely result in deviations in the overall effort associated with an action, defined as the integral of control forces τ_*j,k*_ over the entire *ith* instance of gesture *k*, summed over all *p* mobility space dimensions:

(14) $$\begin{array}{l} e_{i}=\overset{p}{\underset{j=1}{\sum }}\int_{x=0}^{2\pi }\tau_{i,j,k}^{2} \end{array}$$

If *e*_*i*_ is smaller than the smallest value of *e* observed prior to iteration *i*, then the value of $\varepsilon N\left(\mu ,\sigma^{2}\right)$ from the current iteration is added to the kernel weights in Equation (14). Any instance *i* that does not reach the target is considered a failed trial, and is not considered further. This, or a similar constraint on target achievement, is necessary because the “optimal” movement, from this perspective, would otherwise be to remain motionless. Similar constraints have been used in existing optimal control models of speech (Perrier et al., 2005; Patri et al., 2015).

These small deviations in weight, when summed over the course of many trials, will be associated with an overall change in the overall energy expenditure associated with the gestures, and with the overall trajectory of the jaw in task and mobility spaces.

### Example: Jaw Control With Perturbation Adaptation

In order to provide an illustration of these optimization concepts in the domain of speech motor control, we present an example using greatly simplified model of the speech motor system. The example is inspired by the experiments of Tremblay et al. (2003), in which subjects were asked to speak the utterance “see-at” while a velocity-dependent force field was applied to the jaw that induced jaw protrusion. Initially, this caused increased curvature away from the relatively straight-line jaw movements produced as baseline. After a period of exposure, this curvature was reduced and the jaw movements became similar to the movement produced in the absence of the force-field.

**Figure 4:**
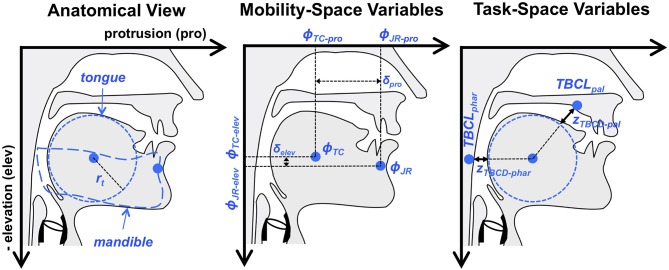
Overview of the articulatory model used for illustrative examples in the present paper. Indicated are the relevant elements of vocal tract anatomy and head-related coordinate system (i.e., protrusion and elevation), mobility variables (i.e., jaw and tongue body position) and task variables (tongue body constriction degree in the pharynx and near the palate). The tongue is assumed to move passively with the jaw. *TBCL*_*phar*_ and *TBCL*_*pal*_ are fixed points in task space that are used, in conjunction with corresponding constriction degree targets, to shape motion patterns in the tongue body constriction variables, *z*_*TBCD*−*phar*_ and *z*_*TBCD*−*pal*_, that create the desired palatal or pharyngeal constrictions.

We model jaw movements as a two degree of freedom system in terms of elevation and protrusion. The dimensions of elevation and protrusion align relatively well with the biomechanical forces applied to the human jaw by orofacial musculature in the relatively restricted range of jaw movements used for speech. They therefore represent a reasonable, if simplified, definition of the mobility space. Making the assumption that the tongue is passively moving in conjunction with the jaw, in this narrow experimental situation, it is also possible to define vocal tract constrictions in the pharyngeal and the palatal regions as higher-level descriptions of the articulatory speech tasks. This conceptualization of jaw movements is shown in Figure 4.

In order to model the relationship between the task (speech gesture) and mobility (jaw movement) spaces, we must ascertain the kinematic relationships between the two. In our simulations, the direct kinematic relationships between the tract variables and mobility variables are:

(15) $$\begin{array}{l} z_{TBCD-phar}\\ =\sqrt{{\left(\phi_{TC-pro}-TBCL_{phar,pro}\right)}^{2}+\;{\left(\phi_{TC-elev}-TBCL_{phar,elev}\right)}^{2}}-r_{t} \end{array}$$

(16) $$\begin{array}{l} z_{TBCD-pal}\\ =\sqrt{{\left(\phi_{TC-pro}-TBCL_{pal,pro}\right)}^{2}+\;{\left(\phi_{TC-elev}-TBCL_{pal,elev}\right)}^{2}}-r_{t} \end{array}$$

where *r*_*t*_ represents the radius of the tongue body, as can be seen in Figure 4. Movement of the tongue center (ϕ_*TC*_), in this example, is affected only by movement of the jaw, as measured at a jaw reference (ϕ_*JR*_) point on the mandible. In other words, the tongue is assumed to move passively with the jaw, such that ϕ_*TC*−*pro*_ = ϕ_*JR*−*pro*_ − δ_*pro*_ and ϕ_*TC*−*elev*_ = ϕ_*JR*−*elev*_ − δ_*elev*_, where δ_*pro*_ and δ_*elev*_ represent the horizontal and vertical offsets, respectively, of ϕ_*TC*_ from ϕ_*JR*_, and are constrained to be constants (see middle panel of Figure 4). Future examples could incorporate independent actuation of the tongue by defining δ_*pro*_ and δ_*elev*_ as mobility variables.

The mobility state variable ϕ is considered to represent the position of the tongue center and jaw reference points in head-related coordinates described by protrusion (i.e., horizontal position relative to the head) and elevation (i.e., vertical position relative to the head). The variables *z*_*TBCD*−*phar*_ and *z*_*TBCD*−*pal*_ are the constriction degree variables for the tongue body, closely related to the Tongue Body Constriction Degree (TBCD) tract variable described in Task Dynamics (e.g., Saltzman and Munhall, 1989).

For the purposes of illustration, the present example maintains two constriction degree variables, each with its own target value. The constriction degree values are defined with respect to corresponding tongue body constriction location (TBCL) targets in the pharyngeal (phar) and palatal (pal) regions of the vocal tract, and represent the Euclidean distance between tongue body center and the given constriction locations minus the radius of the tongue body.

The forward dynamics of the jaw's movement are modeled simply, according to the following equations:

(17) $$\begin{array}{l} {\ddot{\phi }}_{JR-pro}=\left(\tau_{pro}+f_{p}\right)/m_{jaw}, \end{array}$$

(18) $$\begin{array}{l} {\ddot{\phi }}_{JR-elev}=\tau_{elev}/m_{jaw}, \end{array}$$

where *m*_*jaw*_ is the mass of the jaw, and τ_*pro*_ and τ_*elev*_ are the control forces applied to the jaw. To model the velocity-dependent force field, a force (*f*_*p*_) is used to perturb the jaw as it moves:

(19) $$\begin{array}{l} f_{p}=b{\overset{∙}{\phi }}_{JR-elev}, \end{array}$$

where *b* is a constant.

The Jacobian, *J*, is the matrix of first-order partial derivatives of *z* with respect to ϕ_*JR*_:

(20) $$\begin{array}{l} J\left(\phi \right)=\;\left[\begin{array}{cc} \frac{-\left(TBCL_{phar,pro}-\phi_{TC-pro}\right)}{\sqrt{{\left(TBCL_{phar,pro}-\phi_{TC-pro}\right)}^{2}+{\left(TBCL_{phar,elev}-\phi_{TC-elev}\right)}^{2}}} & \frac{-\left(TBCL_{phar,elev}-\phi_{TC-elev}\right)}{\sqrt{{\left(TBCL_{phar,pro}-\phi_{TC-pro}\right)}^{2}+{\left(TBCL_{phar,elev}-\phi_{TC-elev}\right)}^{2}}}\\ \frac{-\left(TBCL_{pal,pro}-\phi_{TC-pro}\right)}{\sqrt{{\left(TBCL_{pal,pro}-\phi_{TC-pro}\right)}^{2}+{\left(TBCL_{pal,elev}-\phi_{TC-elev}\right)}^{2}}} & \frac{-\left(TBCL_{pal,elev}-\phi_{TC-elev}\right)}{\sqrt{{\left(TBCL_{pal,pro}-\phi_{TC-pro}\right)}^{2}+{\left(TBCL_{pal,elev}-\phi_{TC-elev}\right)}^{2}}} \end{array}\right] \end{array}$$

**Figure 5:**
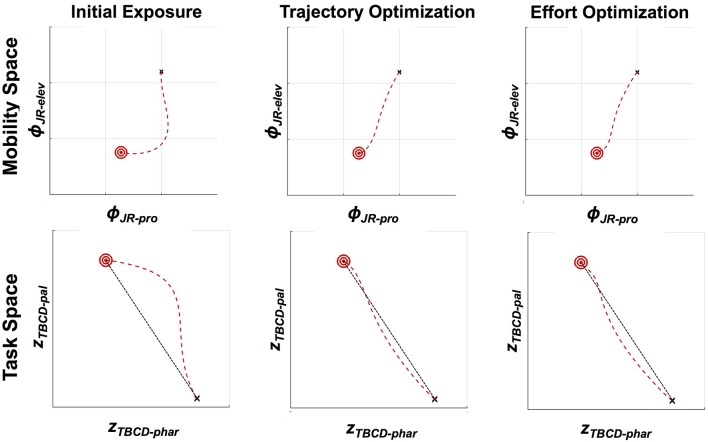
Kinematics of the jaw in mobility (jaw protrusion vs. jaw elevation) and task (pharyngeal constriction degree vs. palatal constriction degree) spaces, showing three different DMP kernel weightings (11 kernels used, with linear spacing in planning space). Starting position is indicated by a black “x,” and the target is indicated by a red bullseye, with the trajectory shown as a red dashed line. Unperturbed jaw motion would lead to a straight-line trajectory from the starting point to the target. With a perturbation of the type described in Equation (19) (*b* = 0.07, *m*_*jaw*_ = 1), the trajectory in both mobility and task space deviates substantially from a straight line. Both optimization schemes lead to approximately straight trajectories, as shown. In the case of trajectory optimization, this is because the optimization is explicitly seeking to reproduce a straight line. In the case of effort minimization, the straight line trajectory emerges as a consequence of lowering the overall control forces applied to the jaw (200 iterations, ε = 0.001).

An example of adaptation to jaw perturbation via optimization of both trajectory tracking and effort minimization is shown in Figure 5. For these simulations, we generate trajectories from /i/ to /ae/ based on the “see-at” trajectories studied in Tremblay et al. (2003). We assume /i/ has as a target a narrow palatal constriction of 0.05 arbitrary units while /ae/ has as a target a wide pharyngeal constriction of 0.3 arbitrary units (Browman et al., 2006). The trajectories generated from both optimization approaches are similar to the trajectories produced after adaptation to the velocity-dependent force field in Tremblay et al. (2003). Both approaches result in a return to fairly straight trajectories in both task and mobility space, which are very similar to the baseline condition. Interestingly, both approaches result in a small initial over-correction for the force field. While we hesitate to read too much into this result given the highly simplified model used in theses simulations, this pattern matches the results seen in arm reaching (Izawa et al., 2008), where the initial over correction has been shown to be the optimal solution to minimize motor effort. A hint of similar patterns for jaw movements can be seen in the data shown in Tremblay et al. (2008), though this is not always seen in the example data shown in these studies (Tremblay et al., 2003; Lametti et al., 2012). Such differences could potentially be attributed to cross-speaker differences in uncertainty about the force field dynamics (Izawa et al., 2008).

## Coordination of Multiple Gestures

One of the benefits of the DMP approach is the use of a separate planning system that governs the activation of the forcing function. We have shown how this same signal can also be used as a “go” cue to gate movement. In this latter sense, the planning system functions in an analogous way to the planning oscillators in Task Dynamics (Saltzman and Byrd, 2000; Saltzman et al., 2008). These planning oscillators, which are themselves dynamical systems, serve to gate the activation of gestures in that model. For example, a gesture might be activated when the phase of the planning oscillator reaches 0°, and be deactivated at a later phase (e.g., 270°). Essentially, we have replaced the planning oscillator from Task Dynamics with our planning system, which controls both the activation of a gesture as well as the evolution of the gesture's associated forcing function.

However, one of the key benefits of the planning oscillators in Task Dynamics has been their additional use in modeling the coordination between separate gestures (Browman and Goldstein, 2000; Nam and Saltzman, 2003; Goldstein et al., 2007, 2009; Saltzman et al., 2008). If we are to replace the planning oscillator with our proposed planning system, we need to ensure that the planning system can also account for this inter-gestural coordination. In order to do this, we will need to slightly amend the planning system dynamics presented earlier.

We start from the assumption that each gesture is associated with its own planning system. However, a planning system with the dynamics in Equation (11), which we have suggested controls the evolution of the control system during movement, is not a good model for planning, since it converges from its initial value of 0 to a multiple of 2π without repeating. While this behavior is useful to control the activation and time course of the control system, it is less useful for replicating the phase coordination between different gestures.

During the planning phase, we assume that the planning systems are, instead, rhythmic dynamical systems with constant phase velocity. It is then possible to allow phase coupling between planning systems in the following way:

(21) $$\begin{array}{l} m_{i}{\dot{x}}_{k}=\alpha_{x}+C_{kl} \end{array}$$

(22) $$\begin{array}{l} \text{where }C_{kl}=\alpha_{kl}\sin\left(\left[x_{k}-x_{l}\right]+\varphi_{kl}\right), \end{array}$$

for two gestures *k* and *l*. The variable φ_*kl*_ denotes the target relative phase between *x*_*l*_ and *x*_*k*_, where their relative phase is defined as *x*_*k*_ − *x*_*l*_. Note that this sine-based coupling term is typical of many papers in the coordination dynamics literature (Haken et al., 1985; Rand et al., 1988; Schmidt et al., 1991, 1993), but differs from the linear coupling term typical of the published literature on DMPs. A linear term may be viewed as a small-angle approximation of sine-based coupling.

Additional coupling terms can be added for additional oscillators that may also be coupled in a multi-way coupling unit. During planning, the planning systems are initiated with a random relative phase. The systems are then allowed to converge to a stable phase relationship. Convergence can be defined in several ways, but is presently defined as:

(23) $$\begin{array}{l} \underset{i}{\sum }\underset{j}{\sum }{Ċ}_{i,j}^{2}<\delta , \end{array}$$

where Ċ_*i,j*_ is the derivative, with respect to time, of the coupling term between gestures *i* and *j*, and δ is the convergence parameter.

After convergence, and upon initiation (triggered at *x*_1_ = 0), the rhythmic planning systems switch to discrete systems, as in Equation (10). Conceptually, the discrete dynamics cause the oscillating planning system to compete a single, final cycle. This transition from rhythmic to discrete dynamics allows us retain the benefits of both rhythmical planning systems, such as stable relative phasing, on the one hand, as well as those of a discrete system for movement control on the other. These include intuitive activation gating, where the planning system triggers movement from its initial value until it converges to its stable final value (c.f. the relatively arbitrary phases for movement gating in the Task Dynamics planning oscillator model), as well as ensuring that the forcing function terminates at the end of the movement (a rhythmic system, such as an underdamped oscillator, would repeat the forcing function). Lastly, the discrete planning system effectively turns itself off when it reaches its convergence value, while planning oscillators would continue to cycle indefinitely.

This model effectively suggests that the planning and movement execution, while both governed by dynamical systems, exhibit different dynamical patterns. Interestingly, this hypothesis receives some support from intracranial recordings made in non-human primates during reaching movements (Churchland et al., 2006, 2010; Shenoy et al., 2013). These studies have shown that both movement planning and movement execution exhibit reliable patterns of neural activity consistent with the evolution of a dynamical system, but that the character of these dynamical patterns is qualitatively different between the two phases, and that this transition can be characterized as a transition between two different network dynamics (Shenoy et al., 2013).

**Figure 6:**
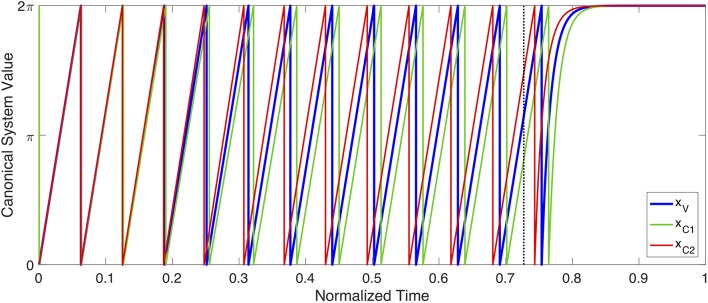
Example of planning system oscillation, coupling and initiation. The state of three separate planning systems (x_V_, x_C1_ and x_C2_) are shown. Both x_C1_ and x_C2_ are coupled in-phase (i.e., φ_*C*1−*V*_ = 0, φ_*C*2−*V*_ = 0) with x_V_, but coupled anti-phase with each other (i.e., φ_*C*1−*C*2_ = −π, φ_*C*2−*C*1_ = π). The three planning systems oscillate across time, according to Equation (21), with their relative phases shifting due to coupling, until they eventually converge on a stable phase pattern. The point of convergence is shown with a dotted, vertical black line. After convergence, each planning system continues to oscillate until it begins a new cycle (i.e., *x* = 0), at which point its associated gesture initiates, and the system becomes governed by Equation (10). The coupling pattern shown here results in gestural initiation in the following order: x_C1_, x_V_, x_C2_. This ordering, and the coupling relationships that produce it, are consistent with the well-studied c-center effect (Browman and Goldstein, 1988), if one interprets the gesture associated with x_V_ as a vowel, and x_C1_ and x_C2_ as onset consonants within the same syllable as that vowel.

As a proof of concept, an example of planning system oscillation, coupling and initiation can be seen in Figure 6. In this simulation, three gestures (C1, C2, and V) are coupled together to form a syllable with a complex onset. C1 and C2 are both coupled in phase with V (φ_C1−V_ = 0, φ_C2−V_ = 0) but anti-phase with each other (φ_C1−C2_ = − π, φ_C2−C1_ = π). During planning, each planning system oscillates with a stable frequency (shown with phase unwrapped in Figure 6). During planning, the three gestures settle into a stable relative phase relationship due to their coupling, with C1 slightly preceding V, which in turn precedes C2. When these relationships converge to become stable, the planning systems switch from rhythmic to discrete dynamics when they next reach a phase 0, triggering initiation of their associated gestures. During movement execution, the discrete dynamics drive each planning system asymptotically toward their final value, 2π. This example demonstrates the capability of this framework to reproduce the well-studied c-center effect (Browman and Goldstein, 2000; Goldstein et al., 2009), where initial consonants in an onset cluster begin before the onset of the syllable's vowel, which in turn precedes the onset of the final consonant in the onset cluster. For example, in the word /spa/, tongue tip movement for the [s] begins before tongue body movement for [a], which in turn begins before lip movement for [p]. The ability of Task Dynamic's planning oscillator model to derive c-center and other patterns of intergestural coordination is a strength of that model, which is maintained in the proposed approach.

## Discussion

We have presented a framework for incorporating principles of optimal control into a dynamical systems framework for modeling the speech motor control system. This was accomplished through the addition of a forcing function to the second-order dynamical system previously hypothesized to regulate speech motor control in the Task Dynamics model. Specifically, this forcing function took a form consistent with the Dynamical Movement Primitives (DMPs) framework, which provides the ability to flexibly modulate the dynamics of the control system. We then showed how the integration of DMPs into the control system allows us to model the observed adaptation to velocity-dependent force fields applied to the jaw during speech production. Importantly, this framework is flexible enough to incorporate a wide variety of features to be optimized and optimization algorithms. We showed how two different approaches can result in similar behavior, by optimizing over dynamic (total force) or kinematic (straight line) criteria. Lastly, we showed how the planning system which governs the temporal unfolding of the control system can also account for the temporal gating of individual speech gestures as well as the temporal organization between separate gestures (such as the c-center effect).

The DMP approach outlined here provides a coherent way to account for speech behavior that is otherwise difficult to reconcile with the dynamical systems approach to speech motor control, while retaining many of the benefits of that approach that have been developed over a long history of research. Importantly, the DMP model may have applications outside the narrow case of jaw perturbations explored here.

First, DMPs can provide a way to model competing constraints on the speech production system, such as the balance between articulatory effort on one hand and target achievement or intelligibility on the other (Lindblom, 1990). Importantly, models based on optimal trajectory control have shown that by changing the relative costs associated with these factors, it is possible to produce speech with varying degrees of target undershoot (Perrier et al., 2005; Patri et al., 2015). These changes may plausibly underlie articulatory changes associated with different speaking conditions and contexts (Lindblom, 1990; Bradlow, 2002). Undershoot can also be modeled in a dynamical systems framework by decreasing the duration of a gesture's activation (Browman and Goldstein, 1992; Parrell, 2011; Parrell and Narayanan, 2018) or by changing the other parameters of the dynamical controller (e.g., mass, damping, and stiffness). However, there is to date no principled way to relate changes in the control system parameters to the hypothesized constraints of effort and intelligibility. DMPs provide this bridge, and could provide a principled way of modeling articulatory changes associated with different speech registers or conditions.

The combination of dynamical systems control with movement optimization via DMPs may also be useful in a number of other areas of speech motor control. For example, it is well-established that articulatory kinematics do not reach stable, adult-like patterns until at least late adolescence (Walsh and Smith, 2002). Such protracted maturation of kinematic patterns could potentially be related to the development of stable forcing functions in the dynamical controller. Additionally, we have shown the DMPs are able to incorporate tracking of explicit trajectories into a dynamical systems framework. Targets with an explicit temporal component have been suggested to be critical for speech (Guenther, 2016). DMPs could thus potentially bridge this seemingly otherwise intractable divide between trajectory- and point-target- based theories. Indeed, it seems highly probable that the auditory target trajectories in DIVA (Guenther, 2016) could be reformulated as dynamical systems with DMPs in an auditory task space. Moreover, DMPs also provide a way to produce trajectories without an explicit time-dependency, which allows them to be more flexible.

Another possible use of our DMP model is in explaining temporo-spatial variation in production across different words. For example, words that are produced more frequently are typically more reduced than less frequent words (Munson and Solomon, 2004). This result is compatible with a stochastic optimization process driven by reinforcement from a listener. For example, the production-driven criteria for a “failed trial,” discussed surrounding Equation (14) (i.e., whether the target was achieved) could be replaced by a reinforcement signal provided by a listener (i.e., they understand what was said). More frequent words would be more likely to be perceived correctly, and so more likely to receive a positive reward signal for any given amount of reduction. Moreover, given a stochastic optimization, more frequent words provide more opportunities for learning, which would lead to a more optimal production. Separately from word frequency, neighborhood density has also been shown to be related to reduction, such that words with more lexical neighbors exhibit less reduction than words of similar frequency with fewer lexical neighbors (Munson and Solomon, 2004). Again, such a pattern could plausibly be generated by a DMP controller, as words with more lexical competition would be more frequently confused by a listener, leading to less positive reinforcement of more reduced productions than in cases where there is little lexical competition. Importantly, such a system would *implicitly* adjust production based on a history of reinforcement, without the need to explicitly include an estimate of a listener's perceptual system (c.f. Lindblom, 1990; Wright, 2004). Alternatively, a more complex criteria that quantifies the degree of articulatory undershoot (Simko and Cummins, 2011) would be able to drive similar behaviors.

The scheme outlined above would imply that different words may be associated with their own (set of) forcing functions. To this point, we have avoided a discussion of the scope of the DMP forcing functions. However, it seems likely that they are associated with higher-level production units, such as words. The evidence for this, aside from the potential utility of the DMPs to model differential effects of word frequency and lexical neighborhood density, comes primarily from studies that have examined the generalizability of learned alterations to speech motor behavior. For example, participants who learned to adapt to a velocity-dependent force filed applied to the jaw failed to transfer this learning to untrained words, even when the patterns of jaw movement were very similar (Tremblay et al., 2008). However, other studies using auditory, rather than force-field, perturbations have shown that learning is somewhat generalizable (Rochet-Capellan et al., 2012). While force-field and sensory-perturbation learning are likely driven by different processes (Krakauer et al., 1999), this suggests that learning or optimization may be occurring at multiple levels of the production hierarchy (gestures, phonemes, syllables, words, etc.). It remains a question for future work to determine the precise nature of how and where optimization may play a role in speech production. However, the notion that words or syllables may be used at the units of speech planning, at least in some cases, is a common idea in many models (Levelt et al., 1999; Kröger et al., 2009; Guenther, 2016).

The DMP approach used here shares some conceptual similarities with the Embodied Task Dynamics model (Simko and Cummins, 2010a,b, 2011). Both approaches augment the basic Task Dynamics model in order to allow for optimization of a cost function. However, the present approach differs from the Embodied Task Dynamics model in a number of critical ways and addresses complementary phenomena. We optimize a forcing function that affects the production of individual gestures at the task level, but that does not change the timing of gestural activation. We show how optimization can be accomplished on the basis of minimizing effort/force, or through the use of an inverse model. The resulting model is shown to account for adaptation to a force-field applied to the jaw. On the other hand, the Embodied Task Dynamics model optimizes the stiffness and activation timing of gestures. This optimization is done of the basis of a cost function that includes terms for articulatory effort, target achievement, and total movement duration. This model has been successful in replicating some important aspects of interarticulator timing and articulatory undershoot. A more thorough comparison of the two approaches is needed in future work.

We have demonstrated that DMPs provide a method for modeling adaptation to altered system dynamics introduced by a novel force field. Such adaptation has alternately been viewed as either the generation of an “internal model” of the system dynamics that can be used to counteract the external forces (Shadmehr and Mussa-Ivaldi, 1994; Krakauer et al., 1999) or as a process of reoptimization of movement to achieve maximum performance (Izawa et al., 2008). We have shown that DMPs are compatible with both views. Given the demonstrated flexibility of the DMP approach (Schaal et al., 2007; Ijspeert et al., 2013), it is likely that it could also be used to adapt to more complex force field dynamics, including time-varying dynamics. However, adaptation in motor performance occurs not only in the presence of novel dynamics but also when alterations are introduced to movement kinematics, such as visuomotor rotations for reaching (Cunningham, 1989; Kagerer et al., 1997; Krakauer et al., 1999; Mazzoni and Krakauer, 2006) or shifts to vowel formants or pitch for speech (Houde and Jordan, 1998; Purcell and Munhall, 2006). Importantly, dynamic and kinematic adaptation have been suggested to be separate processes in human behavior (Krakauer et al., 1999; Rabe et al., 2009; Donchin et al., 2012). Adaptation to kinematic perturbations is typically thought to occur through either changes to “forward models” that predict the sensory consequences of movement (Mazzoni and Krakauer, 2006; Tseng et al., 2007; Shadmehr and Krakauer, 2008) and/or changes to “inverse models” that associate a goal with the motor commands necessary to achieve that goal (Kawato and Gomi, 1992; Wolpert et al., 1995; Wolpert and Kawato, 1998; Kawato, 1999). In our view, it is unlikely that DMPs would provide a satisfactory model for these types of kinematic adaptation. From a theoretical standpoint, neither a forward model (action-sensory mapping) nor inverse model of this type (goal-action mapping) is well-captured by DMPs. Empirically, a critical characteristic of adaptation to kinematic perturbations is that the final, adapted movement remains distinct from the initial, unperturbed movement. This is reflected in a change in reach angle in visuomotor rotation or a change in formant frequencies/vocal tract shape in auditory perturbations. While DMPs are well-suited to model arbitrary trajectories, they retain the (desirable) equifinality of critically-damped, second order dynamical systems. In our view, this means they are likely unable to cause the types of changes seen in kinematic adaptation.

In sum, combining optimal control with dynamical systems in speech motor control holds promise to provide a unified account of a number of different speech behaviors. We have shown that incorporating a tunable forcing function based on Dynamic Movement Primitives provides a way to combine these two separate approaches. Future work is needed to incorporate DMPs into a more plausible model of the speech motor system beyond the simplified jaw system in the current simulations, as well as to test the limits of this approach to explain different aspects of speech behavior.

## Author Contributions

BP and AL conceived the project, developed the theory, implemented the computational models, and wrote the paper.

### Conflict of Interest

The authors declare that the research was conducted in the absence of any commercial or financial relationships that could be construed as a potential conflict of interest.

## Footnotes

^1^Both the FACTS (Parrell et al., 2019) and the ACT (Kröger et al., 2009) models also incorporate dynamical systems control.

^2^The term “mobility space” comes from the robotics literature (Sciavicco and Siciliano, 2000). It is used here rather than the more common “articulatory space” to provide a neutral reference to the kinematic configuration of the vocal tract and avoid confusion over whether “articulatory” refers to a low-level description of the vocal tract geometry (e.g., a concrete description of muscle lengths, or a more abstracted version of vocal tract kinematics such as that provided by the model articulators currently used in TD or in our jaw movement example given below) or a higher-level description of task spaces (such as that provided by the gestural tract-variable space currently used in TD and in our jaw example below).

^3^In the present work, and especially the jaw movement example given below, the mobility space is taken to be an abstracted geometric description of the speech articulator configuration. Such geometric variables are conceptually related to the *model articulators* described in the literature on TD and included as part of the CASY model (Rubin et al., 1996). Currently, neither TD nor our jaw example include a model of the vocal tract's musculature and dynamics. We note that future developments in TD and in our work may implement a muscular mobility space as a replacement for the current abstract geometric variables or, alternatively, use such geometric variables as an intermediate step between task space and the control of muscle activations. Either development could be easily integrated with the present modeling efforts.

^4^Building on the weighted Jacobian pseudo-inverse above, the gating role of the ‘go’ signal was implemented using a diagonal matrix *G* (i.e., a matrix of ‘go’ signals), *J*^*^ = *W*^−1^*GJ*^*T*^(*GJW*^−1^*GJ*^*T*^ + [*I* − *G*])^−1^.

^5^The control and planning systems are called the canonical and output systems, respectively, in previous presentations of DMPs (Schaal et al., 2007; Ijspeert et al., 2013). We have chosen to rename these systems to be consistent with the task-level dynamical control law (Equation 2) and planning oscillators in the Task Dynamics model. We also believe that these names more intuitively reflect these systems' functions.

^6^The range of planning system values (i.e., 0–2π) differs from the literature on DMPs, which use a range of 1–0. This change was made so that the planning system values would be compatible with the model of multi-gesture planning presented below. In that model, the temporal coordination of multiple gestures is accomplished through gestural coupling and entrainment during an oscillator phase preceding the initiation of action, similar to Saltzman and Byrd (2000) and later work (Goldstein et al., 2009; Nam et al., 2009). The discrete planning system described here is conceptualized as a single, final oscillation of those planning oscillators that governs movement execution.

## References

[B1] Beckman M. E. De Jong K. Jun S.-A.-.A. Lee S.-H.-.H. (1992). The interaction of coarticulation and prosody in sound change. Lang. Speech 35, 45–58. 10.1177/002383099203500205 1287391

[B2] Beckman M. E. Edwards J. (1992). Intonational categories and the articulatory control of duration, in Speech Perception, Production, and Linguistic Structure, eds Tohkura Y. Vatikiotis-Bateson E. Sagisaka Y. (Tokyo: Ohmsha, Ltd), 359–375.

[B3] Bernstein N. A. (1967). The Coordination and Regulation of Movements. London: Pergamon Press.

[B4] Bradlow A. R. (2002). Confluent talker- and listener-related forces in clear speech production, in Laboratory Phonology, Vol. 7, eds Gussenhoven C. Warner N. 241–273. Retrieved from: https://www.researchgate.net/publication/265413998_Confluent_Talker-_and_Listener-Related_Forces_in_Clear_Speech_Production

[B5] Browman C. P. Goldstein L. (1988). Some notes on syllable structure in articulatory phonology. Phonetica 45, 140–155. 10.1159/000261823 3255974

[B6] Browman C. P. Goldstein L. (1990). Tiers in articulatory phonology, with some implications for casual speech, in Papers in Laboratory Phonology I: Between the Grammar and Physics of Speech, eds Kingston J. Beckman M. E. (Cambridge: Cambridge University Press), 341–376.

[B7] Browman C. P. Goldstein L. (1992). Articulatory phonology: an overview. Phonetica 49, 155–180. 10.1159/000261913 1488456

[B8] Browman C. P. Goldstein L. (2000). Competing constraints on intergestural coordination and self-organization of phonological structures. Bull. Commun. Parlée 5, 25–34.

[B9] Browman C. P. Goldstein L. Nam H. Rubin P. Proctor M. Saltzman E. (2006). TADA (TAsk Dynamics Application) Manual. New Haven, CT: Haskins Labs.

[B10] Bullock D. Grossberg S. (1988). Neural dynamics of planned arm movements: Emergent invariants and speed-accuracy properties during trajectory formation. Psychol. Rev. 95, 49–90. 10.1037/0033-295X.95.1.49 3281179

[B11] Byrd D. Kaun A. Narayanan S. Saltzman E. (2000). Phrasal signatures in articulation, in Papers in Laboratory Phonology V, eds Broe M. B. Pierrehumbert J. B. (Cambridge: Cambridge University Press), 70–87.

[B12] Byrd D. Saltzman E. (1998). Intragestural dynamics of multiple prosodic boundaries. J. Phon. 26, 173–199. 10.1006/jpho.1998.0071

[B13] Byrd D. Saltzman E. (2003). The elastic phrase: Modeling the dynamics of boundary-adjacent lengthening. J. Phon. 31, 149–180. 10.1016/S0095-4470(02)00085-2

[B14] Churchland M. M. Cunningham J. P. Kaufman M. T. Foster J. D. Nuyujukian P. Ryu S. I.. (2012). Neural population dynamics during reaching. Nature 487, 51–56. 10.1038/nature11129 22722855 PMC3393826

[B15] Churchland M. M. Cunningham J. P. Kaufman M. T. Ryu S. I. Shenoy K. V. (2010). Cortical preparatory activity: representation of movement or first cog in a dynamical machine? Neuron 68, 387–400. 10.1016/j.neuron.2010.09.015 21040842 PMC2991102

[B16] Churchland M. M. Santhanam G. Shenoy K. V. (2006). Preparatory activity in premotor and motor cortex reflects the speed of the upcoming reach. J. Neurophysiol. 96, 3130–3146. 10.1152/jn.00307.2006 16855111

[B17] Cunningham H. A. (1989). Aiming error under transformed spatial mappings suggests a structure for visual-motor maps. J. Exp. Psychol. Hum. Percept. Perform. 15, 493–506. 10.1037/0096-1523.15.3.493 2527958

[B18] Desmurget M. Grafton S. (2000). Forward modeling allows feedback control for fast reaching movements. Trends Cogn. Sci. 4, 423–431. 10.1016/S1364-6613(00)01537-0 11058820

[B19] Diedrichsen J. Shadmehr R. Ivry R. B. (2010). The coordination of movement: Optimal feedback control and beyond. Trends Cogn. Sci. 14, 31–39. 10.1016/j.tics.2009.11.004 20005767 PMC4350769

[B20] Donchin O. Rabe K. Diedrichsen J. Lally N. Schoch B. Gizewski E. R.. (2012). Cerebellar regions involved in adaptation to force field and visuomotor perturbation. J. Neurophysiol. 107, 134–147. 10.1152/jn.00007.2011 21975446

[B21] Edwards J. Beckman M. E. Fletcher J. (1991). The articulatory kinematics of final lengthening. J. Acoust. Soc. Am. 89, 369–382. 10.1121/1.400674 2002175

[B22] Flash T. Hogan N. (1985). The coordination of arm movements: an experimentally confirmed mathematical model. J. Neurosci. 5, 1688–1703. 10.1523/JNEUROSCI.05-07-01688.1985 4020415 PMC6565116

[B23] Goldstein L. Chitoran I. Selkirk E. (2007). Syllable structure as coupled oscillator modes: evidence from Georgian vs. Tashlhiyt Berber, in Proceedings of the XVIth International Congress of Phonetic Sciences, eds Trouvain W. Barry W. (Saarbücken), 241–244.

[B24] Goldstein L. Nam H. Saltzman E. Chitoran I. (2009). Coupled oscillator planning model of speech timing and syllable structure, in Frontiers in Phonetics and Speech Science, eds Fant G. Fujisaki H. Shen J. (Beijng: The Commercial Press), 239–249.

[B25] Guenther F. H. (2016). Neural Control of Speech. Cambridge, MA: The MIT Press.

[B26] Haken H. Kelso J. A. S. Bunz H. (1985). A theoretical model of phase transitions in human hand movements. Biol. Cybern. 347–356. 10.1007/BF00336922 3978150

[B27] Harris M. C. Wolpert M. D. (1998). Signal-dependent noise determines motor planning. Nature 394, 780–784. 10.1038/29528 9723616

[B28] Hoff B. Arbib M. A. (1993). Models of trajectory formation and temporal interaction of reach and grasp. J. Mot. Behav. 25, 175–192. 10.1080/00222895.1993.9942048 12581988

[B29] Hoffmann H. Pastor P. Park D.-H. Schaal S. (2009). Biologically-inspired dynamical systems for movement generation: automatic real-time goal adaptation and obstacle avoidance, in 2009 IEEE International Conference on Robotics and Automation (Kobe), 2587–2592.

[B30] Houde J. F. Jordan I. M. (1998). Sensorimotor adaptation in speech production. Science 279, 1213–1216. 10.1126/science.279.5354.1213 9469813

[B31] Ijspeert A. J. Nakanishi J. Hoffmann H. Pastor P. Schaal S. (2013). Dynamical movement primitives: learning attractor models for motor behaviors. Neural Comput. 25, 328–373. 10.1162/NECO_a_00393 23148415

[B32] Ijspeert A. J. Nakanishi J. Schaal S. (2002). Learning attractor landscapes for learning motor primitives, in Proceedings of the 15th International Conference on Neural Information Processing Systems (Cambridge, MA), 1547–1554. Retrieved from: http://dl.acm.org/citation.cfm?id=2968618.2968810

[B33] Izawa J. Rane T. Donchin O. Shadmehr R. (2008). Motor adaptation as a process of reoptimization. J. Neurosci. 28, 2883–2891. 10.1523/JNEUROSCI.5359-07.2008 18337419 PMC2752329

[B34] Kagerer F. A. Contreras-Vidal J. L. Stelmach G. E. (1997). Adaptation to gradual as compared with sudden visuo-motor distortions. Exp. Brain Res. 115, 557–561. 10.1007/PL00005727 9262212

[B35] Kawato M. (1999). Internal models for motor control and trajectory planning. Curr. Opin. Neurobiol. 9, 718–727. 10.1016/S0959-4388(99)00028-8 10607637

[B36] Kawato M. Gomi H. (1992). A computational model of four regions of the cerebellum based on feedback-error learning. Biol. Cybern. 68, 95–103. 10.1007/BF00201431 1486143

[B37] Kistemaker D. A. Wong J. D. Gribble P. L. (2010). The central nervous system does not minimize energy cost in arm movements. J. Neurophysiol. 104, 2985–2994. 10.1152/jn.00483.2010 20884757

[B38] Kistemaker D. A. Wong J. D. Gribble P. L. (2014). The cost of moving optimally: kinematic path selection. J. Neurophysiol. 112, 1815–1824. 10.1152/jn.00291.2014 24944215 PMC4200004

[B39] Krakauer J. W. Ghilardi M.-F. Ghez C. (1999). Independent learning of internal models for kinematic and dynamic control of reaching. Nat. Neurosci. 2, 1026–1031. 10.1038/14826 10526344

[B40] Kröger B. J. Kannampuzha J. Neuschaefer-Rube C. (2009). Towards a neurocomputational model of speech production and perception. Speech Commun. 51, 793–809. 10.1016/j.specom.2008.08.002

[B41] Lametti D. R. Nasir S. M. Ostry D. J. (2012). Sensory preference in speech production revealed by simultaneous alteration of auditory and somatosensory feedback. J. Neurosci. 32, 9351–9358. 10.1523/JNEUROSCI.0404-12.2012 22764242 PMC3404292

[B42] Lammert A. C. Shadle C. H. Narayanan S. S. Quatieri T. F. (2018). Speed-accuracy tradeoffs in human speech production. PLoS ONE 13:e0202180. 10.1371/journal.pone.0202180 30192767 PMC6128466

[B43] Levelt W. Roelofs A. Meyer A. (1999). A theory of lexical acess in speech production. Behav. Brain Sci. 22, 1–75. 10.1017/S0140525X99001776 11301520

[B44] Lindblom B. (1990). Explaining phonetic variation: a sketch of the H&H theory, in Speech Production and Modelling, eds Hardcastle J. W. Marchal A. (Dordrecht: Kluwer Academic Publisher), 403–439.

[B45] Mazzoni P. Krakauer J. W. (2006). An implicit plan overrides an explicit strategy during visuomotor adaptation. J. Neurosci. 26, 3642–3645. 10.1523/JNEUROSCI.5317-05.2006 16597717 PMC6674132

[B46] Mistry M. Theodorou E. Schaal S. Kawato M. (2013). Optimal control of reaching includes kinematic constraints. J. Neurophysiol. 110, 1–11. 10.1152/jn.00794.2011 23554437

[B47] Munson B. Solomon N. P. (2004). The effect of phonological neighborhood density on vowel articulation. J. Speech Lang. Hear. Res. 47:1048. 10.1044/1092-4388(2004/078) 15605431 PMC4336539

[B48] Nam H. Goldstein L. Saltzman E. (2009). Self-organization of syllable structure: a coupled oscillator model, in Approaches to Phonological Complexity, eds Pellegrino F. Marisco E. Chitoran I. Coupé C. (Berlin; New York, NY: Mouton de Gruyter), 299–328.

[B49] Nam H. Saltzman E. (2003). A competitive, coupled oscillator model of syllable structure, in International Conference on Phonetic Sciences (Barcelona).

[B50] Nashed J. Y. Crevecoeur F. Scott S. H. (2012). Influence of the behavioral goal and environmental obstacles on rapid feedback responses. J. Neurophysiol. 108, 999–1009. 10.1152/jn.01089.2011 22623483

[B51] Nelson W. (1983). Physical principles for economies of skilled movements. Biol. Cybern. 46, 135–147. 10.1007/BF00339982 6838914

[B52] O'Sullivan I. Burdet E. Diedrichsen J. (2009). Dissociating variability and effort as determinants of coordination. PLoS Comput. Biol. 5:e1000345. 10.1371/journal.pcbi.1000345 19360132 PMC2661023

[B53] Parrell B. (2011). Dynamical account of how /b, d, g/ differ from /p, t, k/ in Spanish: evidence from labials. Lab. Phonol. 2, 423–449. 10.1515/labphon.2011.016 23843928 PMC3703669

[B54] Parrell B. Narayanan S. (2018). Explaining coronal reduction: prosodic structure and articulatory posture. Phonetica 75, 151–181. 10.1159/000481099 29433121 PMC5892835

[B55] Parrell B. Ramanarayanan V. Nagarajan S. Houde J. (2019). The FACTS model of speech motor control: fusing state estimation and task-based control. PLoS Comput. Biol. 15:e1007321. 10.1371/journal.pcbi.1007321 31479444 PMC6743785

[B56] Patri J.-F. F. Diard J. Perrier P. (2015). Optimal speech motor control and token-to-token variability: a Bayesian modeling approach. Biol. Cybern. 109, 611–626. 10.1007/s00422-015-0664-4 26497359

[B57] Perrier P. Ma L. Payan Y. (2005). Modeling the production of VCV sequences via the inversion of a biomechanical model of the tongue, in Proceeding of the INTERSPEECH: Interspeech'2005–Eurospeech, 9th European Conference on Speech Communication and Technology (Lisbon), September 4–8, 2005, 1041–1044.

[B58] Purcell D. W. Munhall K. G. (2006). Adaptive control of vowel formant frequency: evidence from real-time formant manipulation. J. Acoust. Soc. Am. 120, 966–977. 10.1121/1.2217714 16938984

[B59] Rabe K. Livne O. Gizewski E. R. Aurich V. Beck A. Timmann D.. (2009). Adaptation to visuomotor rotation and force field perturbation is correlated to different brain areas in patients with cerebellar degeneration. J. Neurophysiol. 101, 1961–1971. 10.1152/jn.91069.2008 19176608

[B60] Rand R. Cohen A. Holmes P. (1988). Systems of coupled oscillators as models of central pattern generators, in Offprints From Neural Control of Rhytmic Movements in Vertebrates, ed Cohen A. (New York, NY: John Wiley & Sons), 333–367.

[B61] Rochet-Capellan A. Richer L. Ostry D. J. (2012). Nonhomogeneous transfer reveals specificity in speech motor learning. J. Neurophysiol. 107, 1711–1717. 10.1152/jn.00773.2011 22190628 PMC3311670

[B62] Rubin P. Saltzman E. Goldstein L. McGowan R. Tiede M. T. Browman C. P. (1996). CASY and extensions to the task-dynamic model, in Proceedings of the 4th Speech Production Seminar (Autrans).

[B63] Saltzman E. (1986). Task dynamic coordination of the speech articulators: a preliminary model. Exp. Brain Res. Series 15, 129–144. 10.1007/978-3-642-71476-4_10

[B64] Saltzman E. (1999). Nonlinear dynamics of temporal patterning in speech, in Proceedings of Symposium on the Dynamics of the Production and Perception of Speech, A Satellite Symposium of the XIVth International Congress of Phonetic Sciences (Berkeley, CA), eds Divenyi P. L. Porter R. J..

[B65] Saltzman E. Byrd D. (2000). Task-dynamics of gestural timing: Phase windows and multifrequency rhythms. Hum. Mov. Sci. 19, 499–526. 10.1016/S0167-9457(00)00030-0

[B66] Saltzman E. Kelso J. A. S. (1987). Skilled actions: a task dynamic approach. Psychol. Rev. 94, 84–106. 10.1037/0033-295X.94.1.84 3823306

[B67] Saltzman E. Munhall K. G. (1989). A dynamical approach to gestural patterning in speech production. Ecol. Psychol. 1, 333–382. 10.1207/s15326969eco0104_2

[B68] Saltzman E. Nam H. Krivokapić J. Goldstein L. (2008). A task-dynamic toolkit for modeling the effects of prosodic structure on articulation, in Proceedings of the Speech Prosody 2008 Conference (Campinas), 175–184.

[B69] Schaal S. Mohajerian P. Ijspeert A. J. Cisek P. Drew T. Kalaska J. F. (2007). Dynamics systems vs. optimal control a unifying view. Progr. Brain Res. 165, 425–445. 10.1016/S0079-6123(06)65027-9 17925262

[B70] Schmidt R. C. Beek P. J. Treffner P. J. Turvey M. T. (1991). Dynamical substructure of coordinated rhythmic movements. J. Exp. Psychol. Hum. Percept. Perform. 17, 635–651. 10.1037/0096-1523.17.3.635 1834782

[B71] Schmidt R. C. Shaw B. K. Turvey M. T. (1993). Coupling dynamics in interlimb coordination. J. Exp. Psychol. Hum. Percept. Perform. 19, 397–415. 10.1037/0096-1523.19.2.397 8473847

[B72] Sciavicco L. Siciliano B. (2000). Modelling and Control of Robot Manipulators. London: Springer.

[B73] Sergio L. E. Scott S. H. (1998). Hand and joint paths during reaching movements with and without vision. Exp. Brain Res. 122, 157–164. 10.1007/s002210050503 9776514

[B74] Shadmehr R. Krakauer J. W. (2008). A computational neuroanatomy for motor control. Exp. Brain Res. 185, 359–381. 10.1007/s00221-008-1280-5 18251019 PMC2553854

[B75] Shadmehr R. Mussa-Ivaldi F. A. (1994). Adaptive representation of dynamics during learning of a motor task. J. Neurosci. 14, 3208–3224. 10.1523/JNEUROSCI.14-05-03208.1994 8182467 PMC6577492

[B76] Shenoy K. V. Sahani M. Churchland M. M. (2013). Cortical control of arm movements: a dynamical systems perspective. Annu. Rev. Neurosci. 36, 337–359. 10.1146/annurev-neuro-062111-150509 23725001

[B77] Simko J. Cummins F. (2010a). Embodied task dynamics. Psychol. Rev. 117, 1229–1246. 10.1037/a0020490 21038977

[B78] Simko J. Cummins F. (2010b). Sequencing embodied gestures in speech. Adv. Cognit. Syst. 71:301 10.1049/PBCE071E_ch11

[B79] Simko J. Cummins F. (2011). Sequencing and optimization within an embodied task dynamic model. Cogn. Sci. 35, 527–562. 10.1111/j.1551-6709.2010.01159.x

[B80] Stavisky S. D. Willett F. R. Murphy B. A. Rezaii P. Memberg W. D. Miller J. P. (2018). Neural ensemble dynamics in dorsal motor cortex during speech in people with paralysis. BioRxiv 505487. 10.1101/505487 PMC6954053 31820736

[B81] Tilsen S. (2013). A dynamical model of hierarchical selection and coordination in speech planning. PLoS ONE 8:e62800. 10.1371/journal.pone.0062800 23638147 PMC3634742

[B82] Todorov E. (2004). Optimality principles in sensorimotor control. Nat. Neurosci. 7, 907–915. 10.1038/nn1309 15332089 PMC1488877

[B83] Todorov E. Jordan M. I. (2002). Optimal feedback control as a theory of motor coordination. Nat. Neurosci. 5, 1226–1235. 10.1038/nn963 12404008

[B84] Tremblay S. Houle G. Ostry D. J. (2008). Specificity of speech motor learning. J. Neurosci. 28, 2426–2434. 10.1523/JNEUROSCI.4196-07.2008 18322088 PMC6671181

[B85] Tremblay S. Ostry D. (2006). The achievement of somatosensory targets as an independent goal of speech production-special status of vowel-to-vowel transitions, in Dynamics of Speech Production and Perception, eds Divenyi P. Greenberg S. Meyer G. (Amsterdam: IOS Press), 33–43.

[B86] Tremblay S. Shiller D. Ostry D. (2003). Somatosensory basis of speech production. Nature 423, 866–869. 10.1038/nature01710 12815431

[B87] Tseng Y.-W. W. Diedrichsen J. Krakauer J. W. Shadmehr R. Bastian A. J. (2007). Sensory prediction errors drive cerebellum-dependent adaptation of reaching. J. Neurophysiol. 98, 54–62. 10.1152/jn.00266.2007 17507504

[B88] Uno Y. Kawato M. Suzuki R. (1989). Formation and control of optimal trajectory in human multijoint arm movement. Biol. Cybern. 61, 89–101. 10.1007/BF00204593 2742921

[B89] Walsh B. Smith A. (2002). Articulatory movements in adolescents: evidence for protracted development of speech motor control processes. J. Speech Lang. Hear. Res. 45, 1119–1133. 10.1044/1092-4388(2002/090) 12546482

[B90] Wolpert D. M. Ghahramani Z. Jordan M. I. (1995). An internal model for sensorimotor integration. Science 269, 1880–1882. 10.1126/science.7569931 7569931

[B91] Wolpert D. M. Kawato M. (1998). Multiple paired forward and inverse models for motor control. Neural Netw. 11, 1317–1329. 10.1016/S0893-6080(98)00066-5 12662752

[B92] Wright R. (2004). Factors of lexical competition in vowel articulation, in Phonetic Interpretation: Papers in Laboratory Phonology VI, eds Local J. Ogden R. Temple R. (Cambridge: Cambridge University Press), 26–50.

